# Relationship between early musical training and detection of binaural gap based on interaural correlation change

**DOI:** 10.3389/fnins.2025.1580045

**Published:** 2025-06-17

**Authors:** Mengyuan Wang, Senlan Hu, Jinjun Liu, Mei Ai, Lingzhi Kong

**Affiliations:** ^1^Beijing Key Laboratory of Applied Experimental Psychology, National Demonstration Center for Experimental Psychology Education, Beijing Normal University, Beijing, China; ^2^Faculty of Psychology, Beijing Normal University, Beijing, China; ^3^Kunming Child and Adolescent Mental Health Service Centre, Yunnan, China; ^4^Cognitive Science and Allied Health School, Institute of Life and Health Sciences, Key Laboratory of Language and Cognitive Science, Ministry of Education, Beijing, China

**Keywords:** musical training, auditory, binaural, interaural correlation, sensitive period

## Abstract

**Introduction:**

The auditory fusion of binaural sounds and the perceived auditory image are determined by the similarities of the sounds at the two ears. Sensitivity to the change in interaural correlation, a measure of interaural similarity, is crucial to extract target sound from noisy background. Although musicians have been found to perform better than non-musicians in various types of auditory processing tasks such as frequency discrimination or temporal resolution, the relationship between musical training and the interaural correlation processing remains poorly understood.

**Methods:**

Here we embedded a fragment of interaurally uncorrelated noises (interaural correlation = 0) into the interaurally identical marker noises (interaural correlation = 1) and constructed a binaural gap based on the change in interaural correlation (from 1 to 0 then back to 1). The minimum duration of interaurally uncorrelated fragment for detecting the binaural gap (duration threshold) was determined for groups of young adults without musical training and those who started musical training early (before 7 years of age) or late (after 8 years of age).

**Results:**

When the binaural noises arrived simultaneously (Experiment 1), we found that the duration threshold was significantly correlated with the onset age of musical training for the early-trained musicians but no such significant correlation was observed for the late-trained musicians. Moreover, the duration thresholds for the early-trained musicians were significantly shorter than those for both the late-trained musicians and non-musicians. When interaural delay was introduced (Experiment 2), this early-musical-training-related enhancement in interaural correlation processing was maintained for binaural noises when the interaural delay was 2 ms, while no enhancement was found when the interaural delay was 4 ms.

**Discussion:**

Our findings suggest that sensitivity to dynamic changes in interaural correlation might be influenced by musical training in early childhood, implying a sensitive period when musical training has a significant impact on interaural correlation processing.

## Introduction

Musical training has a profound influence on auditory processing. Musicians outperformed non-musicians across a range of auditory tasks, including frequency discrimination (Boebinger et al., 2015; Deguchi et al., 2012; Kyrtsoudi et al., 2023; Lee et al., 2020; Madsen et al., 2019; Meha-Bettison et al., 2018; Parbery-Clark et al., 2009; Spiegel and Watson, 1984), temporal resolution (Kumar et al., 2014; Kumar et al., 2016; Pirouzmand et al., 2022; Rammsayer and Altenmüller, 2006; Ryn Junior et al., 2022; Zendel and Alain, 2012) and speech recognition against noisy background (Başkent and Gaudrain, 2016; Bidelman and Yoo, 2020; Clayton et al., 2016; Coffey et al., 2017; Du and Zatorre, 2017; Hennessy et al., 2022; Kaplan et al., 2021; Kyrtsoudi et al., 2023; Li et al., 2021; Meha-Bettison et al., 2018; Parbery-Clark et al., 2009; Puschmann et al., 2019; Swaminathan et al., 2015; Zendel and Alain, 2012; Zhang et al., 2021). Musicianship-related enhancement for the processing spectral and temporal information have also been observed at both the cortical (Hao et al., 2023; Jünemann et al., 2023; Li et al., 2018; Pantev et al., 1998; Puschmann et al., 2021) and subcortical levels (Musacchia et al., 2007; Parbery-Clark et al., 2012; Tierney et al., 2015; Wong et al., 2007).

Critically, the timing of training onset plays a pivotal role in shaping these enhancements. For example, Pantev et al. (1998) found that the auditory cortical representation of piano tones in early-adulthood musicians was significantly correlated with the age at which they began training. Similarly, Bailey and Penhune (2012) reported a correlation between the age of training onset and behavioral performance in an auditory-motor rhythm synchronization task (RST). Further, Bailey and Penhune (2013) identified a non-linear relationship between training onset age and RST performance, with a critical shift around ages 7–9. When musicians are divided into early-trained (onset ≤ 7 years) and late-trained (onset > 7 years) groups, better performance was consistently found for early-trained group compared with the late-trained group and non-musicians (Bailey and Penhune, 2010, 2012, 2013; Bailey et al., 2014; Penhune et al., 2005; Vaquero et al., 2016; Watanabe et al., 2007), suggesting a sensitive period for the effect of musical training.

Beyond monaural processing (e.g., frequency discrimination and temporal resolution), binaural processing, i.e., the fusion and localization of sounds from both ears into a unified auditory object, relies on comparing inputs between the left and right ears (Cherry and Sayers, 1956; Cherry and Taylor, 1954). In recent years, the relationship between musical training and binaural processing has received more attention. Musicians were found to have superior binaural processing abilities in discrimination of interaural time and level differences (Madsen et al., 2019; Nisha et al., 2022; Nisha et al., 2023; Wright and Dai, 2024) and other studies showed that musicians were more adept at discriminating interaural phase differences than non-musicians (Bianchi et al., 2019; Ding et al., 2022; Pirouzmand et al., 2022). Notably, Wright and Dai (2024) found that early-trained musicians outperformed non-musicians in discrimination of interaural level difference but not interaural time difference. However, no studies to date have explored the relationship between musical training and interaural coherence processing, a fundamental mechanism for sound localization and speech-in-noise perception (Stern and Trahiotis, 1995; Licklider, 1948; Durlach et al., 1986).

Interaural correlation, one measure of interaural coherence, is defined as the maximum cross-correlation coefficient of the two sound waves at the left and right ears after one sound has been time shifted (Grantham, 1995). Dynamic processing of the interaural correlation can be assessed using the binaural gap detection paradigm (Akeroyd and Summerfield, 1999). In this paradigm, listeners detect a brief segment of binaurally independent noise (interaural correlation: 0) embedded in coherent noise (correlation: 1). The minimum detectable duration of this gap—the duration threshold—reflects sensitivity to dynamic changes in interaural coherence (Boehnke et al., 2002; Kong et al., 2012; Kong et al., 2015). The interaural correlation processing is strongly affected by interaural delay. For binaurally identical noises, human listener perceives a fused sound image when the interaural delay was 0 ms while the image becomes completed diffused for interaural delay of a few milliseconds (Blodgett et al., 1956; Mossop and Culling, 1998). Our previous studies showed that duration thresholds increase with interaural delay (Kong et al., 2012, 2015) and the maximal interaural delay for binaural gap detection was associated with speech in noise recognition under simulated reverberant conditions (Li et al., 2013). Intriguingly, Ding et al. (2022) determined the maximum interaural delay for detecting a binaural gap with a fix duration, however, the performance of musicians and non-musicians was similar (Ding et al., 2022).

The current study is going to address two questions: First, does musical training enhance binaural gap detection when sounds arrive simultaneously or with short interaural delays (2–4 ms)? Second, is this enhancement specific to individuals who began training during early sensitive periods? To answer these questions, we examined the duration thresholds for binaural gap detection in three participant groups: early-trained musicians (early-trained group), late-trained musicians (late-trained group) and non-musicians (control group) when the binaural noises arrived at the two ears simultaneously (Experiment 1) and with interaural delays of 2 ms or 4 ms (Experiment 2).

## Materials and methods

### Experiment 1

#### Participants

Forty-one individuals between the ages of 17 and 25 (Mean = 20.0, SD = 2.2) took part in this study. All participants were undergraduate or graduate students from universities in the Beijing vicinity. Participants claimed that they were neurologically healthy and the experience of musical training, including the onset age of training, the duration of training, and whether musicians still kept training was also determined through a questionnaire that was developed within our laboratory. Among the 41 participants, 27 were professional musicians (19 females and 8 males) and 14 were non-musicians (10 females and 4 males). The non-musicians had no more than 1 year of formal music training, excluding compulsory music courses during school education, and belonged to the control group. All musicians had at least 8 years of training experience (Mean = 12.7, SD = 2.3) and were currently undergoing continuous training. Those who began music training before age 7 were classified into the early-trained group (*n* = 15), and those who began at or after age 8 were classified into the late-trained group (*n* = 12). The selection criteria for the onset ages of the early-trained and late-trained groups were similar to previous studies (Bailey and Penhune, 2013; Steele et al., 2013).

Participants’ pure-tone thresholds were no larger than 20 dB HL for frequencies from 0.125 to 8 kHz and their binaural hearing was balanced in that the differences between the pure-tone thresholds for the two ears were less than 15 dB at each frequency. All participants have provided written informed consent, with parental proxy signatures for underage participants. Additionally, all participants have received compensation. All the experiments in this study were approved by the Ethics and Bioethics Committee of Beijing Normal University (BNU202401250008).

#### Apparatus and stimuli

The participant sat on a chair at in a sound attenuated chamber. The experimental stimuli were Gaussian white noise synthesized using the “randn()” function in MATLAB (the MathWorks Inc., Natick, MA, USA). The Gaussian wideband noises (0–10 kHz) had a duration of 1000 ms (rise/fall time: 30 ms). The sampling rate was 48 kHz and the amplitude quantization was 16-bit.

For binaurally identical noises, the noise in the right ear were copied from the left ear (interaural correlation = 1). In order to construct a binaural gap, a segment in the temporal middle of noise for the right ear was substituted by another segment of noise that was binaural independent to the corresponding part of noise for the left ear (binaural gap: interaural correlation = 0). The noises at the two ears always started simultaneously (interaural time delay = 0 ms). Notice that the energy and spectrum of the binaural noises are not changed by the insertion of the binaural gap while the auditory image is modified, i.e., the perceptual compactness/diffuseness, number, placement (Bilsen and Goldstein, 1974; Edmonds and Culling, 2009). If only the sound at one ear was delivered, any changes during the noise could not be detected.

These binaural noises were delivered using a Creative Sound Blaster G6 (Creative SB1770, Creative Technology Ltd., Singapore) and headphones (ATH-MSR7, Audio-Technica Co., USA). Sound intensity was calibrated to 60 dB SPL using an Audiometer (Model 831, Larson Davis, Depew, NY, USA).

#### Procedure

We used a two-interval, two-alternative, forced-choice adaptive procedure. The noises with binaural gap were randomly assigned to one interval and binaurally identical noises was delivered to another interval. The two intervals were separated by 1000 ms.

When the participant arrived at the lab, we asked the participant to wear the headphones and listen to the stimulus with and without the binaural gap. When the sound with the binaural gap was delivered, we asked the participant to report if a change of sound image occurred during the sound. If the participant was able to report the change of sound image, the participant was asked to tell which of the two intervals contained the binaural gap. All participants completed a practice stage to discriminate the binaural noises with and without binaural gap, in order to ensuring that they understood the tasks and requirements before the formal test.

During the threshold measurement, we manipulated the gap duration using a three-down-one-up procedure (Levitt, 1971). If the participant consecutively identified the correct interval with the binaural gap for three times, the gap duration was decreased while the gap duration was increased if the participant made just one incorrect response. The manipulation of the gap duration started at 33 ms and the step size of the change started at 16 ms. Every time the gap duration changed from increase to decrease or from decrease to increase, the step size was reduced by a factor of 0.5 until reaching 1 ms. If the participant made the wrong selection, visible feedback (a red square) would be displayed on the computer screen.

We terminated the test procedure when 12 reversals were reached and used the last 6 reversals to calculate the duration threshold (arithmetic mean). Each participant completed 4 runs of test and we used the best three duration threshold to calculate the duration threshold for this participant.

#### Data analysis

The experimental data were recorded using Presentation (Version 20.1, Neurobehavioral Systems, Inc., Berkeley, CA, USA). Statistical analysis was conducted using SPSS (Version 27.0, IBM Corp, Armonk, NY, USA). The relationship between the duration or onset age of musical training and duration threshold was tested by the Pearson correlation analysis. The effect of musical training and interaural delay on the duration threshold was tested by the repeated-measures ANOVA. There were two independent variables: one is the interaural delay (0 ms, 2 ms, 4 ms), another is the participant groups (musical-training group and the control group, or early-trained group, late-trained group and control group). For each interaural delay, the effect of musical training on the duration threshold was tested by the one-way ANOVA. The effect of musicians’ onset age while controlling training duration was examined by a hierarchical regression analysis with the musicians only. Step 1 included training duration as a control variable, and step 2 included onset age as an independent variable.

### Experiment 2

#### Participants

Fifteen early-trained musicians, 12 late-trained musicians, and 14 non-musicians participated in Experiment 2 who also took part in Experiment 1.

#### Apparatus and stimuli

We used similar binaural-gap stimuli in Experiment 1 except interaural delays were introduced. We added a quiet segment (2 ms or 4 ms) to the beginning of the noise at the right ear and the end of the noise at the left ear. There were two conditions for interaural delay: 2 ms and 4 ms.

#### Procedure

The procedure in Experiment 2 was the same as that in Experiment 1. The duration thresholds for the two interaural-delay conditions (2 ms, 4 ms) were determined. A practice session was also completed before the formal test.

#### Data analysis

Same as Experiment 1.

## Results

### Experiment 1

When there was no interaural delay (0 ms), all participants perceived fused noise at a position in the middle of the head for binaurally identical noises and were able to detect the binaural gap. Therefore, data from all participants were included in the statistical analysis.

**FIGURE 1:**
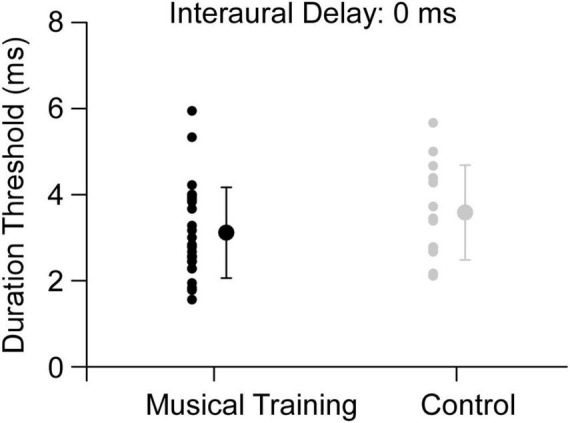
Duration thresholds for musical-training and control group (interaural delay = 0 ms). The dark filled circle represents the individual data and mean for the musical training group and the gray filled circle represents the individual data and mean for the non-musicians. Error bars show standard deviation for each group.

**FIGURE 2:**
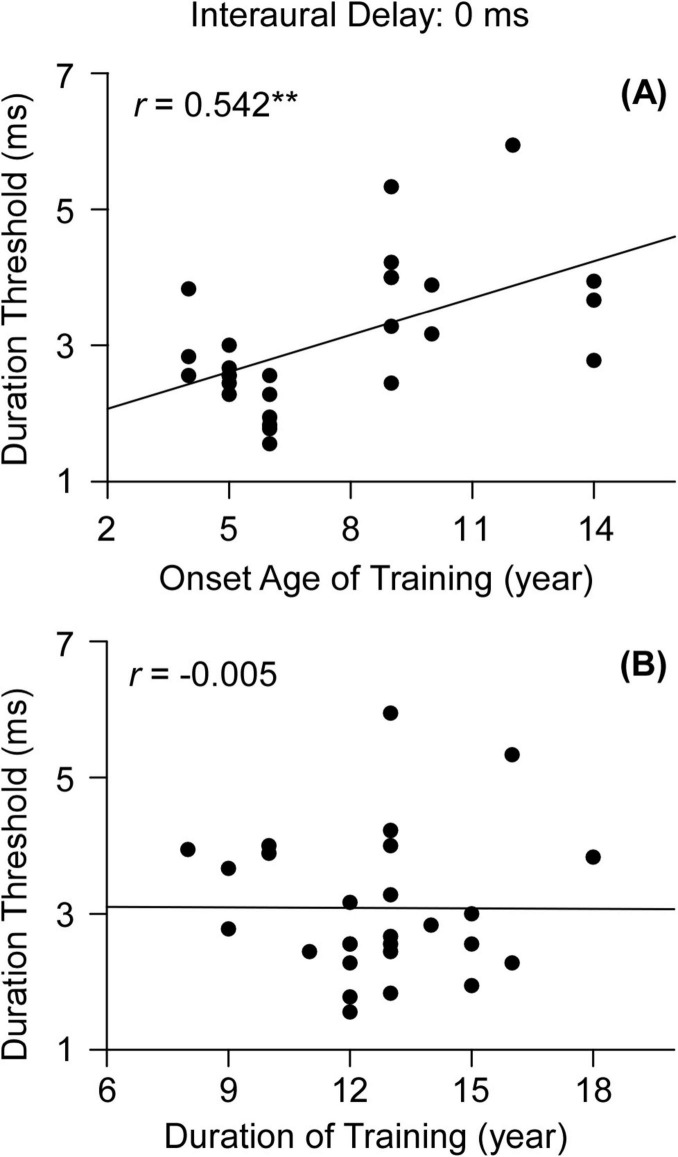
**(A)** Correlation between onset age of training and the duration thresholds. **(B)** Correlation between the duration of musical training and duration thresholds. Significant differences are marked with asterisks (**p < 0.01).

First, we performed a Mann-Whitney U test between all the participants with musical training (musical-training group) and those without (control group). The result showed that the duration threshold of musical-training group was not significantly different from that of control group (*p* = 0.151, Figure 1). Then, we tested the relationship between the duration or onset age of musical training and the duration thresholds for the binaural gap detection. We found the onset age of musical training was significant correlated with the duration threshold (*r* = 0.542, *p* < 0.01, 95% CI [0.194, 0.760], *n* = 27, Figure 2A). In contrast, no significant correlation was found between the duration of musical training and duration threshold (*r* = −0.005, *p* = 0.979, 95% CI [−0.384, 0.376], *n* = 27, Figure 2B).

**FIGURE 3:**
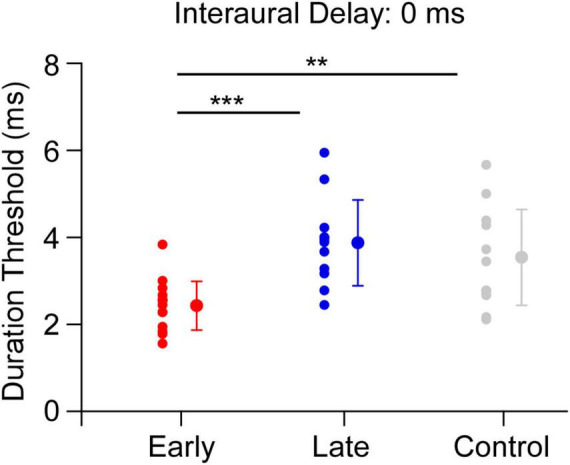
Duration thresholds for early-trained group, late-trained group and control group (interaural delay = 0 ms). The red filled circle represents the individual data and mean for the early-trained group, the blue filled circle represents the individual data and mean for the late-trained group and the gray filled circle represents the individual data and mean for the non-musicians. Error bars show standard deviation for each group (**p < 0.01, ***p < 0.001).

**FIGURE 4:**
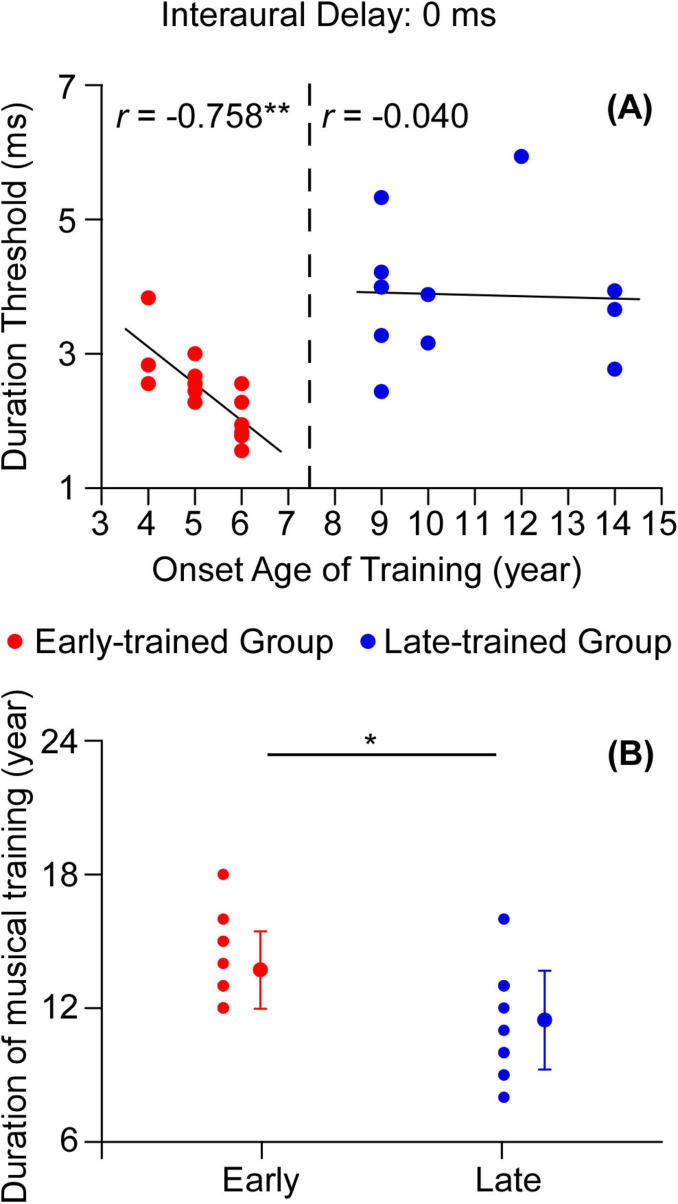
**(A)** Correlation between the onset age of musical training and duration thresholds for early-trained group (red circles) and late-trained group (blue circles) (interaural delay = 0 ms). **(B)** Duration of training for early-trained group (red bar) and late-trained group (blue bar). Significant differences are marked with asterisks (*p < 0.05, **p < 0.01).

We further analyzed the relationship between the onset age of musical training and the duration thresholds for the early-trained and late-trained groups, respectively. A one-way ANOVA was then performed to test the effect of participant group (early-trained group, late-trained group and control group) on the duration threshold. The result showed that the main effect of group was significant [*F*(2,38) = 9.804, *p* < 0.001, eta squared: 0.340, Figure 3]. The Bonferroni *post hoc* test showed that the duration thresholds for early-trained musicians were significantly shorter than those for non-musicians (*p* < 0.01) and the late-trained musicians (*p* < 0.001), while the difference in the duration threshold between late-trained musicians and non-musicians was not significant (*p* = 1.000). In addition, there was a significant correlation between the onset age and duration threshold for the early-trained group (*r* = −0.758, *p* < 0.01, 95% CI [−0.911, −0.380], *n* = 15) while no significant correlation was found between the onset age and duration threshold for the late-trained group (*r* = −0.040, *p* = 0.902, 95% CI [−0.599, 0.548], *n* = 12, Figure 4A).

As the duration of training for the early-trained group was significantly longer than that of the late-trained group [*t*(25) = 2.845, *p* < 0.05, Cohen’s *d*: 2.04, Figure 4B], further analysis was necessary to clarify the effect of the onset age of musical training on the binaural gap detection. We analyzed the prediction of the onset age on musical-training-related improvement in performance using hierarchical multiple regression while removing the role of training duration. The regression shows that the final model was statistically significant [*R*^2^ = 0.542, *F*(2,24) = 14.195, *p* < 0.001]. The addition of the onset age improved the explanation of the dependent variable by 54.2%. Thus, after controlling for the contribution of training duration, the onset age still had a significant effect on predicting the musician’s performance on the task (Table 1).

**TABLE 1:** Hierarchical models of onset age and predictive value of the age of onset beyond duration of musical training to duration threshold of interaural correlation when the interaural delay is 0 ms (B is the regression coefficient and beta is the standardized regression coefficient; *p < 0.05, **p < 0.01, ***p < 0.001).

Variables	Model 1		Model 2	
	***B***	**β**	***B***	**β**
Constants	3.117*		−3.440*	
Duration	−0.002	−0.005	0.313**	0.682
Age of onset			0.336***	1.007
*R* ^2^	0.000		0.542	
Δ*R*^2^	0.000		0.542	
*F*	0.001		14.195***	

### Experiment 2

**FIGURE 5:**
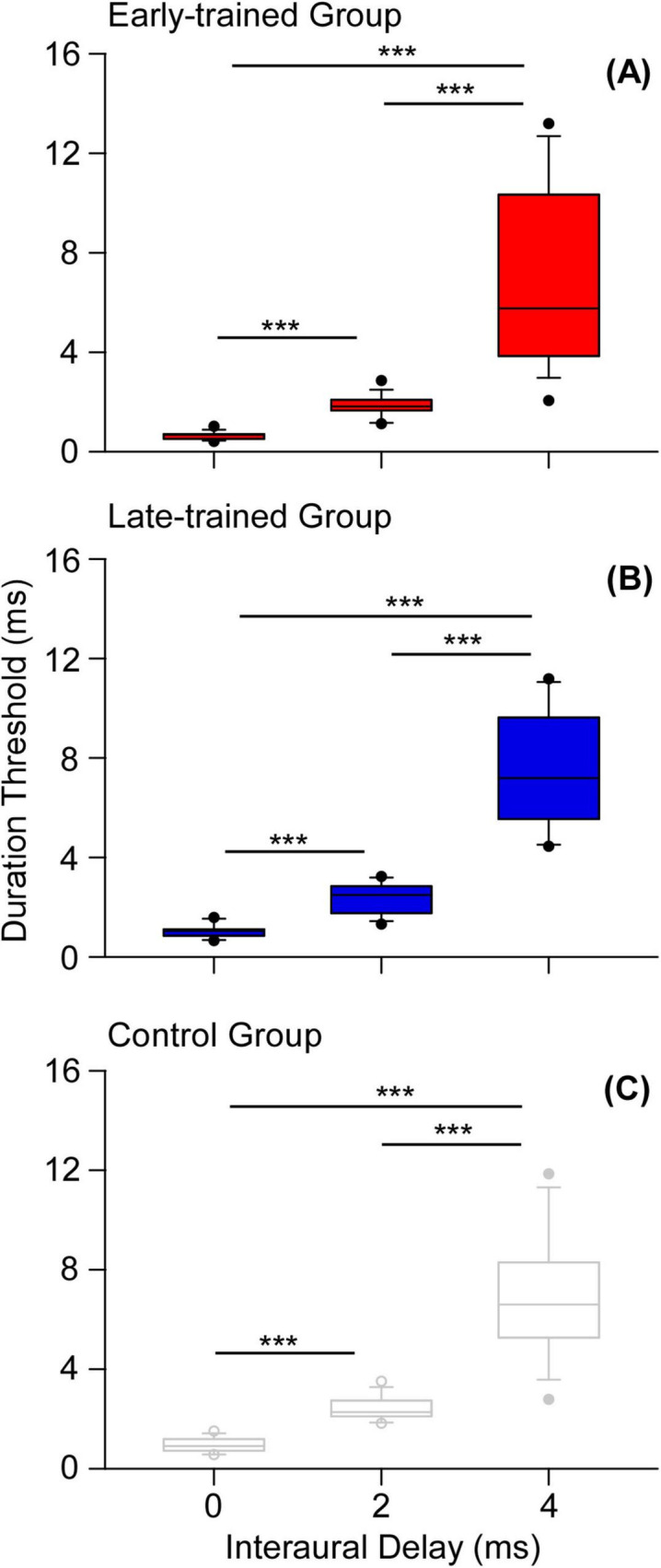
Effect of interaural delay on the duration thresholds for early-trained group **(A)**, late-trained group **(B)** and control group **(C)**. Significant differences are marked with asterisks (***p < 0.001).

First, the effect of interaural delay (2 ms, 4 ms) on detecting the binaural gap was tested for the early-trained, late-trained and control groups. Because the participants in Experiment 2 were identical to those in Experiment 1, the data from Experiment 1 (interaural delay = 0 ms) were also included. A repeated-measures ANOVA, with one within-subject factor (interaural delay: 0, 2, 4 ms) and one between-subject factor (early-onset group, late-onset group and control group) showed that the main effect of interaural delay was significant [*F*(2,76) = 169.626, *p* < 0.001, eta squared: 0.817]. The Bonferroni *post hoc* test showed that the duration thresholds when the interaural delay was 0 ms were significantly shorter than those when the interaural delay was 2 ms (*p* < 0.001) and when the interaural delay was 4 ms (*p* < 0.001). The duration thresholds when the interaural delay was 2 ms were also significantly shorter than those when the interaural delay was 4 ms (*p* < 0.001, Figure 5). The effect of the group was not significant [*F*(2,38) = 1.023, *p* = 0.369, eta squared: 0.051] and the interaction between these two factors was also not significant [*F*(4,76) = 0.143, *p* = 0.874, eta squared: 0.007].

**FIGURE 6:**
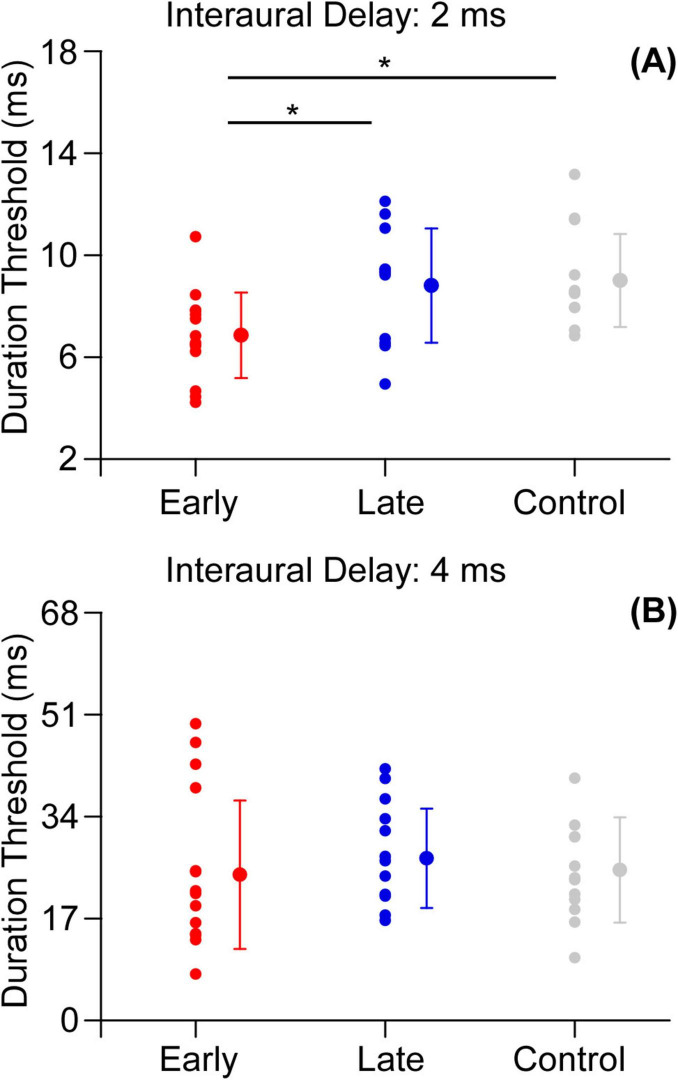
**(A)** Duration thresholds for early-trained group, late-trained group and control group (interaural delay = 2 ms). **(B)** Duration thresholds for early-trained group, late-trained group and control group (interaural delay = 4 ms). The red filled circle represents the individual data and mean for the early-trained group, the blue filled circle represents the individual data and mean for the late-trained group and the gray filled circle represents the individual data and mean for the non-musicians. Error bars show standard deviation for each group (*p < 0.05).

Similar to Experiment 1, a one-way ANOVA was performed to test the effect of participant group (early-trained group, late-trained group and control group) on the duration threshold when the interaural delay was 2 ms and 4 ms, respectively. When the interaural delay was 2 ms, there was a significant main effect of group [*F*(2,38) = 5.597, *p* < 0.01, eta squared: 0.228, Figure 6A]. The Bonferroni *post hoc* test showed that the early-trained musicians outperformed both the non-musicians (*p* < 0.05) and the late-trained musicians (*p* < 0.05). There was no significant difference in duration threshold between late-trained musicians and non-musicians (*p* = 1.000). In contrast, the main effect of group was not significant [*F*(2,38) = 0.250, *p* = 0.780, eta squared: 0.013] when the interaural delay was 4 ms (Figure 6B).

**FIGURE 7:**
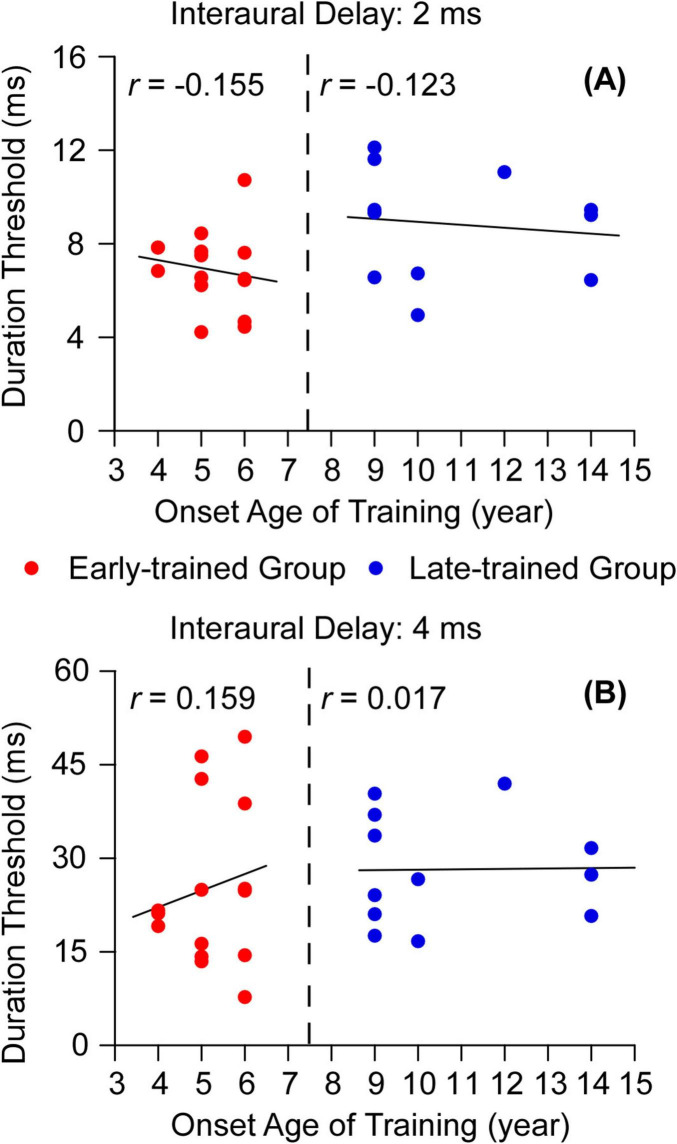
**(A)** Correlation between the onset age of musical training and duration thresholds for early-trained group (red circles) and late-trained group (blue circles) (interaural delay = 2 ms); **(B)** Correlation between the onset age of musical training and duration thresholds for early-trained group (red circles) and late-trained group (blue circles) (interaural delay = 4 ms).

The relationship between the onset age of training and duration threshold was tested for the early-trained group and late-trained group. The result showed that there was no significant correlation between the onset age of musical training and the duration threshold no matter for the early-trained group (*r* = −0.155, *p* = 0.581, 95% CI [−0.615, 0.393], *n* = 15) or for the late-trained group (*r* = −0.123, *p* = 0.703, 95% CI [−0.648, 0.489], *n* = 12) when the interaural delay was 2 ms (Figure 7A). Similarly, when the interaural delay was 4 ms, there was also no significant correlation between the duration threshold and the onset age of musical training either, no matter for the early-trained groups (*r* = 0.159, *p* = 0.571, 95% CI [−0.389, 0.617], *n* = 15) or for the late-trained group (*r* = 0.017, *p* = 0.959, 95% CI [−0.563, 0.584], *n* = 12, Figure 7B).

We also used hierarchical regression to analyze the effect of onset age on musicians’ task performance when the interaural delay was 2 ms after removing the factor of training duration. The procedures were exactly the same as those in Experiment 1. The results demonstrated that the final model (Model 2) included both onset age and training duration with marginal statistical significance [*R*^2^ = 0.187, *F*(2,24) = 2.755, *p* = 0.084]. When the onset age was included, the explanation of the dependent variable increased by 18.4% (Table 2). This result implies that even after controlling for training duration, differences in task performance across musicians who began training at various ages remained when the interaural delay was 2 ms.

**TABLE 2:** Hierarchical models of onset age and predictive value of the age of onset beyond duration of musical training to duration threshold of interaural correlation when the interaural delay is 2 ms (*B* is the regression coefficient and beta is the standardized regression coefficient; *p < 0.05, **p < 0.01).

Variables	Model 1		Model 2	
	***B***	**β**	***B***	**β**
Constants	8.378**		0.607	
Duration	−0.048	−0.052	0.325	0.349
Age of onset			0.398*	0.587
*R* ^2^	0.003		0.187	
Δ*R*^2^	0.003		0.184	
*F*	0.067		2.755	

## Discussion

Our study was to investigate the relationship between musical training and auditory processing of dynamic changes in interaural correlation when there was no interaural delay (Experiment 1) or there were interaural delays (Experiment 2).

### Relationship between musical training and binaural gap detection with no interaural delay

First, the relationship between musical training and binaural gap detection was discussed when all the musicians were included in one group. There was no significant difference in the task performance between the musicians and non-musicians in group level (Figure 1), which implies that musical training might not be a critical factor for binaural gap detection when the interaural delay is 0 ms. This finding partly agrees with that of Ding et al. (2022), who determined the maximum interaural delay at which a binaural gap of fixed duration could be detected and found no significant difference between participants with and without musical training. However, we observed a significant correlation between the onset age of training and detection of binaural gap (Figure 2A) while the detection of binaural gap was not significantly correlated with the duration of musical training (Figure 2B). Considering the previous evidences that early-trained musicians outperformed late-trained musicians of several auditory-motor tasks (Bailey and Penhune, 2010, 2012, 2013), we divided the musicians to an early-trained group and late-trained group and did further analysis for the duration thresholds among three participant groups.

Second, the effect of musical training was compared among three groups: early-trained group, late-trained group and control (non-musicians). We found that early-trained musicians had significantly shorter duration thresholds for binaural gap detection that those for non-musicians and late-trained musicians when the interaural time delay was 0 ms (Figure 3). In contrast to Ding et al. (2022), the results of our study provided the first evidence for the relationship between musical training and interaural correlation processing. Considering the musical-training-related improvement in sensitivity to interaural correlation change was found only in early-trained musicians but not in late-trained musicians, our results support the notion of a sensitive period for the impact of music training on interaural correlation processing. A sensitive period of musical training before age of 7 has been shown on the synchronization performance of visuomotor rhythm (Penhune et al., 2005; Watanabe et al., 2007) and the auditory-motor rhythm (Bailey and Penhune, 2010; Bailey et al., 2014), and also the temporal precision in their piano performance (Vaquero et al., 2016).

In the physiological level, previous studies revealed increased white matter integrity in the corpus callosum (Steele et al., 2013; Schlaug et al., 1995) and enhanced gray matter volume in the ventral premotor cortex (Bailey et al., 2014), which facilitate auditory-motor integration and interhemispheric communication. Early training also strengthens striatal-cortical connectivity (van Vugt et al., 2021) and refines cerebellar-putaminal circuits (Vaquero et al., 2016), supporting precise temporal resolution. These neuroplastic changes align with our behavioural findings, as such networks mature rapidly before age 7 (Skoe and Kraus, 2013; Penhune, 2011), creating a window for experience-dependent optimization. Moreover, electrophysiological evidence from deaf children who received cochlear implants (CIs) showed abnormal cortical response latencies if they received CI stimulation after 7 years of age (Sharma and Campbell, 2011) which suggests the sensitive period of auditory cortex comes to an end at 7 years old. As auditory cortex was shown to play an important role in interaural correlation processing (Chait et al., 2005), it looks reasonable for us to find that the musical training before the age of 7 has more significant impacts on interaural correlation processing compared with musicians who started the training later.

Third, the correlation between the onset age of musical training and binaural gap detection was tested for the early-trained and late-trained groups. For the early-trained group, a significant correlation was observed between the age of onset of musical training and duration thresholds under the condition of 0 ms interaural delay. In contrast, no significant correlation was observed in the late-trained group (Figure 4A). This finding was supported by finding from Bailey and Penhune (2013) that age of onset was correlated with sensorimotor-synchronization performance for early-trained musicians but not for the late-trained musicians. In contrast to the positive correlation found by Bailey and Penhune (2013), the duration thresholds were negatively correlated with the onset age of musical training for the early-trained group in our study (Figure 4A), which indicates that duration thresholds decrease with increasing age of onset training in the early-trained period. Note that the negative correlation between the onset age of musical training and duration thresholds for the early-trained group was also opposite to the positive correlation between these two factors for the entire musician group. A non-linear quadratic fit might be appropriate to explain the relationship between onset age of musical training and duration thresholds for the musical training group, which potentially supporting a “sensitive period” hypothesis. We used a quadratic model for the effect of the onset age and found that the quadratic model’s adjusted *R*^2^ value (0.45) is higher than the linear model’s (0.30). The quadratic coefficient (onset-age^2^) is statistically significant (*p* < 0.05). The critical age of ∼8.2 (vertex of the quadratic curve) years suggests a non-linear relationship, supporting a “sensitive period”.

This negative correlation could be explained in the neural physiological development of the human auditory system. It has been found that neurons in the superior olivary complex, inferior colliculus and auditory cortex encoded the interaural correlation (Coffey et al., 2006; Joris and Yin, 2007). The auditory cortex was found to be important for the processing of the interaural correlation change in human beings (Chait et al., 2005). In contrast to the early maturation for the auditory brainstem before 1 year old (Moore et al., 1995), the auditory cortex develops much slower and the mature thalamocortical axons fill the deeper cortical layers by age 5–6 years (Moore and Guan, 2001). Thus, it is reasonable to propose that the effect of musical training on binaural gap detection may be constrained by the immaturity of auditory cortex development in musicians who began training at a very young age, such as 4 and 5 years old and those started at age 6 might benefit from the musical training because their auditory cortex is more mature for the processing of the interaural correlation and their age was still in the sensitive period.

### Relationship between musical training and binaural gap detection with interaural delays

First, the binaural gap detection was strongly affected by interaural delay. We found that the duration thresholds for binaural gap detection became longer as the interaural delay increased from 0 to 4 ms, no matter for musicians or non-musicians (Figure 5). This finding was in consistent with the findings from our previous studies (Kong et al., 2012; Kong et al., 2015). The reason is that the sound image of noises with an interaural correlation of 1 became more and more diffuse when the interaural delays increased from 0 to several milliseconds (Blodgett et al., 1956; Mossop and Culling, 1998). Our previous study also proved that the increase of interaural delay and decrease of interaural correlation of the marker similarly affected the detection of binaural gap, which implied a trade-off between the decrease of interaural correlation and increase of interaural delay on interaural correlation processing (Kong et al., 2015). Thus, the increase of interaural delay makes the auditory system harder to perceive the difference between the binaural gap and the marker in which binaural gap was embedded.

Second, the relationship between musical training and binaural gap detection was discussed when there were interaural delays. Similar to the findings when there was no interaural delay, we found that the early-trained group still retained some advantage when the interaural delay was 2 ms. Early-trained musicians significantly outperformed late-trained musicians and non-musicians in the detection of binaural gap (Figure 6A). However, the musical-training-related improvement in interaural correlation processing was not observed when the interaural delay was 4 ms (Figure 6B). Oure results partially agree with the finding of Ding et al. (2022) that there was no significant difference between binaural gap detection for musicians and non-musicians when the interaural time delay was extremely large, i.e., more than 10 ms.

### Limitations of the current study

We should note that there are some limitations of the current study. First, our sample size was relatively small which may compromise the reliability of the correlation analysis. For example, the sample size for the late-trained group is 12 and future studies with larger sample size are needed to confirm the absence of a correlation between the onset age and duration thresholds. Moreover, whether early-trained musicians retain a long-term advantage in auditory skills after stopping music training remains to be investigated. Recently, it has been found that former musicians who had given up music training exhibited an advantage in prosodic pitch perception (Toh et al., 2023). In the future, it will be interesting to determine whether former musicians who began music training during a sensitive period and have ceased training still show better ability in interaural correlation processing.

In summary, our findings demonstrate while the interaural correlation processing was strongly affected by interaural delay, early-trained musicians had better performance than the non-musicians. However, late-trained musicians did not show such benefit. Our findings imply that there is a sensitive period for the musical training on the interaural correlation processing.

## Data Availability

The raw data supporting the conclusions of this article will be made available by the authors, without undue reservation.
